# Editorial: From classical breeding to modern biotechnological advancement in horticultural crops - trait improvement and stress resilience, volume II

**DOI:** 10.3389/fpls.2025.1598544

**Published:** 2025-04-07

**Authors:** Pankaj Kumar, Mohammed Wasim Siddiqui, Mohammad Irfan

**Affiliations:** ^1^ Department of Biotechnology, Dr. Yashwant Singh Parmar University of Horticulture and Forestry, Nauni, India; ^2^ Department of Food Science and Postharvest Technology, Bihar Agricultural University, Banka, Bihar, India; ^3^ School of Integrative Plant Science, College of Agriculture and Life Sciences, Cornell University, Ithaca, NY, United States

**Keywords:** fruit, vegetable, horticulture, stress tolerance, sequencing, genetic improvement, QTL, biotechnology

The global challenge of securing food and nutrition against the backdrop of climate change demands urgent and innovative approaches to agricultural practices. As the planet faces increasing environmental pressures, the need for robust and resilient crops that can withstand these changes is more critical than ever (Irfan et al., 2023). Issues such as reduced yields, poor quality, and increased vulnerability to pests and diseases threaten global food systems, requiring urgent intervention. In this context, the transition from classical breeding methods to modern biotechnological advancements has revolutionized the approach to horticultural crop improvement (Kumar et al., 2023). This Research Topic, “From Classical Breeding to Modern Biotechnological Advancement in Horticultural Crops - Trait Improvement and Stress Resilience, Volume II,” brings together 14 research articles and one review that highlight significant strides in improving horticultural crops resilience, quality, and nutritional content through genetic innovations. This is the second volume of our Research Topic on the transformative journey of crop breeding, specifically targeting trait improvement and resilience under stress conditions. Historically, horticultural crops such as fruits, vegetables, and ornamental plants have played an essential role in human nutrition and environmental enhancement. The classical methods of breeding, primarily involving hybridization, mutation breeding, and selection, were fundamental in improving key traits such as yield, size, and disease resistance. However, these methods often faced limitations due to their time-consuming nature and the complexity of the genetic mechanisms involved. The need for faster, more precise improvements led to the development and application of molecular genetics and biotechnological tools.

## Trait improvement

The application of biotechnology has led to substantial progress in the improvement of traits related to yield, quality, and stress resilience. The genetic basis of key horticultural traits plays a fundamental role in crop improvement. Fruit color, a major determinant of consumer preference, is influenced by specific genetic loci. Feng et al. utilized BSA-seq technology to identify quantitative trait loci (QTLs) governing green and mature fruit color in pepper. They found that premature green and pale-green colors were controlled by loci on chromosomes 1 and 10, while mature fruit color was regulated by a recessive allele on chromosome 6. This genetic insight facilitates marker-assisted breeding to develop pepper varieties with desirable fruit color characteristics. Another essential trait in horticultural crops is the bright green leaf (BGL) trait, which enhances the commercial appeal of Chinese kale. Zhang et al. mapped and cloned the *BoBGL* gene using BSR-Seq and molecular marker analysis. The candidate gene, *BoCER1.C8*, was associated with wax synthesis, and variations in its promoter region influenced leaf color expression. Their findings provide a foundation for breeding new Chinese kale varieties with superior leaf aesthetics and market value.

Likewise, in the case of fruit size, studies such as Pan et al. using quantitative trait loci sequencing (QTL-seq) in jujube have identified key genes associated with fruit size, which will be crucial for future breeding programs aimed at optimizing fruit production. Candidate intervals for jujube fruit size were primarily located on chromosomes 1, 5, and 10, with chromosome 1 being the most frequent. QTL-seq and ANNOVAR analysis identified 40 candidate genes from 424 SNPs and 164 InDels, including 37 annotated genes in the jujube genome (Pan et al.). These advancements, coupled with sustainable horticultural practices can meet the increasing demand for food while adapting to changing environmental conditions. Another exciting avenue of research is the exploration of genetic resistance to biotic stresses. Singh et al. have made remarkable progress in identifying genotypes of sponge gourd with stable resistance to Tomato Leaf Curl New Delhi Virus (ToLCNDV), demonstrating the potential for molecular breeding to combat viral diseases that threaten crop yields (Singh et al.). In addition to enhancing yield and resistance, improving the nutritional quality of crops has become a focal point. Wang et al. explored the role of pollen manipulation in modifying seed dormancy in *Paris polyphylla* var. *yunnanensis*, a Chinese medicinal herb. This exhibits cyclic anther opening and closing, influencing seed dormancy and germination through temperature-dependent methylation and gene expression changes. Transcriptomic and methylation analyses identified key dormancy-regulating genes (*IAAS, SUS, GA2ox*, *ABI2, ARP*), with inhibited anther closure promoting dormancy and low-temperature protection facilitating germination *via* altered expression in oxidative phosphorylation, hormone signaling, and zeatin biosynthesis pathways. This study is significant as it paves the way for improving seed viability and nutrient content, contributing to the development of crops with enhanced nutritional profiles (Wang et al.).

## Stress resilience

One of the most pressing challenges faced by horticultural crops is their vulnerability to environmental stresses, including drought, salinity, extreme temperatures, and diseases (Sharma et al., 2022). Traditional breeding methods often fall short in developing varieties with the required level of resilience under these harsh conditions. In contrast, biotechnological interventions, such as the identification and deployment of stress-resilient genes, offer a promising solution. Enhancing crop resilience against biotic stress is critical for sustainable horticulture. Bacterial speck disease, caused by *Pseudomonas syringae* pv. tomato (Pst), threatens tomato production worldwide. Hassan et al. identified resistance to a *virulent Pst* race 1 strain in *Solanum pimpinellifolium* LA1589, revealing three QTLs and five candidate genes associated with disease resistance. This discovery supports molecular marker-assisted breeding efforts to develop tomatoes with durable resistance to bacterial speck (Hasan et al). Similarly, soybean cyst nematode (SCN) poses a major challenge to common bean production. Shi et al. conducted a genome-wide association study (GWAS) and identified multiple QTLs conferring resistance to different SCN HG types. Notably, resistance was more dominantly associated with Mesoamerican rather than Andean domestication. Their findings provide valuable SNP markers for genomic selection, enabling the breeding of SCN-resistant common bean cultivars. Zhou et al. identified the *CmoFL1* gene in pumpkin, which regulates fruit length, offering insights into how genetic variation can be leveraged to improve fruit morphology under varying environmental conditions. In a similar vein, Jia et al. demonstrated how aerospace mutagenesis in tea trees led to enhanced photosynthetic capacity and stress resilience, with changes in hormone metabolism contributing to improved yield. Aviation mutagenesis enhances tea tree growth, photosynthesis, and resistance by increasing key hormones (L-tryptophan, indole, salicylic acid, and salicylic acid 2-O-β-glucoside), which correlate positively with growth indices and yield. Exogenous application of these hormones further boosts leaf area, chlorophyll content, and enzymatic activity, highlighting aviation mutagenesis as a promising method for improving tea cultivation, though its impact on rhizosphere soil requires further investigation. The application of such advanced breeding techniques can fast-track the development of climate-resilient crops capable of thriving in adverse conditions (Jia et al.). Similarly, Albornoz et al. investigated the role of *CBF1* genes in tomatoes to enhance fruit tolerance to chilling stress. Ectopic overexpression of *ShCBF1* and *SlCBF1* in transgenic tomatoes, driven by the *RD29A* promoter, exacerbated postharvest chilling injury (PCI), leading to ripening failure, reduced soluble solids, altered volatile profiles, and increased decay (Albornoz et al.). Transcriptomic analysis indicated disrupted ripening and stress response pathways, while wild-type seedlings from chilled fruit exhibited acclimation, suggesting a potential cold-stress memory effect absent in transgenic lines.

## Bridging the gap- sustainable practices and biotechnological integration

While biotechnology plays a crucial role in improving crop traits and resilience, it must be integrated with sustainable agricultural practices to ensure long-term success. The development of climate-resilient crops requires a multi-faceted approach that combines genetic innovations with sustainable farming techniques (Kumar et al., 2023). This synergy can create agricultural systems that are both productive and environmentally friendly. The importance of this integrated approach is highlighted in the work of Kelimujiang et al., who examined the *WRKY* gene family in *Lavandula angustifolia* (*LaWRKY*), a key plant in the essential oils industry. The *LaWRKY* plays key roles in growth, stress responses, and secondary metabolism, with 207 members identified through bioinformatics analysis. Phylogenetic analysis classified *LaWRKY* into three groups, with segmental duplications driving gene family expansion. Tissue-specific expression revealed 12 genes highly active in flower buds and calyx, suggesting involvement in terpenoid biosynthesis. Environmental stressors regulated *LaWRKY* expression, with light and cold inducing flower bud expression, while drought primarily affected leaves. Overexpression of *LaWRKY57* and *LaWRKY75* in tobacco altered glandular trichome density and volatile terpenoid composition, highlighting their potential regulatory role. Such research not only enhances crop quality but also contributes to the development of sustainable practices for plant-based industries (Kelimujiang et al.).

Controlled environment agriculture (CEA) presents opportunities to enhance crop productivity while minimizing environmental impact. Bhattarai et al. highlighted the potential of genomics-informed breeding, marker-assisted selection, and precision breeding to develop cultivars optimized for CEA systems. Their review underscores the need for breeding strategies tailored to indoor farming environments to ensure economic viability and resource efficiency. Biotechnological advancements also enable rapid and precise trait selection in horticultural crops. Zhao et al. introduced a machine vision-based system for detecting key traits in shiitake mushroom caps, utilizing deep learning and traditional image processing techniques. Their method significantly improved phenotypic assessment efficiency, reducing labor-intensive manual measurements. Such innovations can accelerate breeding programs by facilitating high-throughput phenotyping. Metabolite profiling further enhances our understanding of crop quality traits. In wine grapes, Xia et al. identified the cytochrome P450 enzyme *VvCYP76F14* as a key regulator of wine bouquet precursor production. Through site-directed mutagenesis, they demonstrated that specific amino acid substitutions significantly altered enzymatic activity. Their study provides a potential molecular marker for selecting grape varieties with desirable aromatic profiles, bridging traditional breeding with modern metabolic engineering (Xia et al.). Another example of integrating omics approaches is the study on Gongju (a traditional medicinal herb). Zhao et al. conducted transcriptome and metabolome analyses to investigate chlorogenic acid (CGA) biosynthesis in autooctoploid Gongju. Gongju, exhibits higher chlorogenic acid (CGA) content in autooctoploid compared to tetraploid varieties across flowering stages. Integrated transcriptome and metabolome analyses revealed that key enzymes (pma6460, mws0178) and genes (*CmHQT*, *CmC3H*) involved in CGA synthesis were more highly expressed in octoploid Gongju. Additionally, transcription factors such as *CmMYBs* and *CmbHLHs* play a crucial role in regulating CGA biosynthesis. Such findings contribute to the development of high-value medicinal crops through targeted breeding and biotechnological interventions. By harnessing genomic tools, phenotyping innovations, and sustainable cultivation practices, horticultural crop improvement can achieve new levels of resilience and quality. Bridging traditional breeding with modern biotechnological advancements will be pivotal in meeting future agricultural demands.

Overall, the research presented in this Research Topic highlights the transformative role of biotechnology in modern crop improvement. From enhancing resilience to optimizing nutritional content, the future of horticultural crops lies in the synergy between classical breeding methods and modern genetic innovations. These efforts will pave the way for the development of crops that not only survive but thrive under the changing conditions of our planet, ensuring food and nutritional security for all. We appreciate *Frontiers in Plant Science* for hosting this Research Topic and extend our gratitude to all contributors and reviewers for their valuable insights.
